# Hydration Kinetics for Alkaline Activation of Slag from Color Variation Data

**DOI:** 10.3390/molecules26123764

**Published:** 2021-06-21

**Authors:** Zhaoyang Ding, Jinghai Zhou, Qun Su, Hong Sun, Yichao Zhang, Qing Wang, Hongguang Bian, Fengxin Dong

**Affiliations:** 1 School of Civil Engineering, Shenyang Jianzhu University, Shenyang 110168, China; dingzhaoyang@sjzu.edu.cn (Z.D.); suqun@stu.sjzu.edu.cn (Q.S.); sunhong@sjzu.edu.cn (H.S.); zhangyichao@sjzu.edu.cn (Y.Z.); 2 School of Material Science and Engineering, Shenyang Jianzhu University, Shenyang 110168, China; wangqingmxy@sjzu.edu.cn (Q.W.); bianhg@stu.sjzu.edu.cn (H.B.); dongfx@stu.sjzu.edu.cn (F.D.)

**Keywords:** alkali-activated slag, hydration kinetics, color variation, average pixel value, Krstulovic–Dabic kinetic model

## Abstract

In this study, we explore a new method based on color variation data to derive the kinetics of the entire process of the hydration of alkali-activated slag (AAS). Using this image analysis technique, we can monitor the induction period that cannot be observed using conventional microcalorimetry techniques. Color variation was recorded across a sequence of 9999 images, which were processed via MATLAB software package. Further, an average pixel value (APV) was determined to represent the color in each image. Reaction parameters, such as color variation velocity *v(t)*, reaction speed *ε*(*t*)*,* and hydration degree *α*(*t*), that govern the entire hydration process were determined. On the basis of the reaction parameters and a Krstulovic–Dabic kinetic model, integral and differential equations were derived to simulate the three basic processes of AAS hydration. Equations describing the reaction kinetics of AAS with solutions of three different concentrations of NaOH were extracted using this method.

## 1. Introduction

This study aims to explore the possibility of modeling the hydration kinetics of alkaline activated slag (AAS) from color variation data. As is well known, research on chemical reaction kinetics was initiated in the late nineteenth century. When studying the inversion of cane sugar in the presence of acids, Wilhelmy discovered that the rate of the process was proportional to the residual amount of cane sugar. Following this, scholars such as Guldberg and Waage, van’t Hoff, and Arrhenius made significant contributions to our understanding of chemical reaction kinetics, and the kinetics equation of a generalized homogeneous reaction was established as follows [1]:

d*c*/d*t* = *k*(*T*)*f*(*c*) (1)

where *t* is the time, *c* is the concentration of the product, *k*(*T*) is the temperature dependence of the rate constants, *f*(*c*) is the reaction mechanism function, for which the definition is assumed to be *f*(*c*) = (1 − *c*)*^n^* for a homogeneous reaction system.

This concept was introduced into solid-state kinetics by Lewis in 1905 as follows [1]:

d*α*/d*t* = *k*(*T*)*f*(*α*). (2)

Thus, a concept used to model a heterogeneous solid system was borrowed from a theory developed for a homogeneous system. Because the state of a reacting solid cannot be characterized by its concentration, it was replaced with the extent of conversion, *α*, which corresponds to the hydration degree in a cementing material system [2]. The basic kinetic equations describing AAS chemistry are based on variations in the hydration degree relative to the time elapsed (*α–t* data), where α is the most important parameter. 

Microcalorimetry is a typical method employed to measure the hydration degree of an inorganic cementing material [3]; determine the reaction process and hydration kinetics of AAS [4] and other “soil cements,” such as metakaolin [5], and fly ash-based geopolymers [6]; and examine the effects of various parameters on these cementitious materials, such as reaction temperature [7], alkali type, concentration [8,9], and the nature of the starting precursor [10,11]. However, it should be stressed that cementitious material systems are primarily investigated via kinetics studies that are based on measuring overall physical properties, such as hydration heat. This implies that the direct data measured and used to determine the hydration kinetics do not represent the real hydration degree, *a*, but are based on thermal data; this is presumed to be reasonable because the hydration of AAS is an exothermic phenomenon. Is the hydration heat equal to the hydration degree? Could hydration heat uniquely represent the state of a solid that is engaged in a multi-step reaction? It is difficult to come to an exact conclusion. However, bearing in mind the complicated mineral composition of the slag and the numerous and inconclusive reaction mechanisms and chemical reactions of AAS [12,13,14,15], hydration heat is still used to characterize and calculate the reaction kinetics. However, data entirely extracted from the monitoring of the exothermic process cannot be used to calculate the hydration kinetics because the reaction between slag and alkaline solutions is initially rapid and intense, making it difficult to observe the first exothermic peak (initial period), as it lasts for a few minutes and is caused by the partial dissolution of the slag. Moreover, during the calculation of the hydration kinetics of AAS or cement, only one exothermic peak is required to deduce the key parameters, such as *n*, the reaction degree of nucleation and crystal growth (NG), and the rate constant, *K*, of the hydration kinetics. This is because the value of *n*, for instance, is graphically determined on the basis of the slope of the log–log plot of −ln(1 − *α*) and elapsed time *t*. Thus, the values of *n* and *K* can only be deduced when the log–log plot is a straight line. In other words, these values cannot be calculated when more than one exothermic peak exists. The current method neglects the data at the beginning, during the initial period; that is, the hydration kinetics of AAS/cement as determined via microcalorimetry are incomplete. 

During the hydration of AAS, its color changes from gray to blue. A Japanese scholar in 1957 believed that the color change was caused by the sulfide in the slag. Minute amounts of sulfide such as MnS are generated during the hydration process, which causes the product to exhibit a green–blue color. Some scholars regard this phenomenon as a consequence of valence changes in iron ions [16]. Wang et al. [17] immersed cement-slag paste into a 5% (NH_4_)_2_SO_4_ solution and found that the surface color of the paste faded away. This was explained as a result of the Ca^2+^ inside the cement-slag paste reacting with SO_4_^2−^ in the solution and forming CaSO_4_·2H_2_O, which meant Ca^2+^ affected the color of the product. In fact, there are several explanations regarding the color change phenomena of the slag hydration process. However, no consensus has been reached regarding this metachromatism. However, from the commencement of AAS hydration, with the passage of time, it is observed that the mechanical properties of AAS gradually improve, heat is continually released, and the color continues to become a more intense shade of green–blue. Because heat data can be used to estimate *a* in the complicated multi-step reaction of AAS, it is worthwhile attempting to calculate the AAS kinetic equation using color variation data. In this study, instead of thermal data, *a*, for the AAS hydration process is represented by the color variation of AAS in a constant area, as measured over a long period. By this method, the entire early hydration process of AAS is described, and relevant mathematical modeling and kinetic equations are established and tested.

## 2. Materials and Methods

### 2.1. Materials and Instruments

Blast furnace slag (BFS) from Angang, China, was used in this study; its chemical composition is detailed in Table 1. The specific surface area measured by N_2_ sorption using the Brunauer–Emmett–Teller (BET) method was 427 m^2^/kg. The BFS was activated with a modulus-adjusted water glass. The products used to prepare the solution were laboratory reagents, including 98% pure NaOH pellets supplied by Techcomp Chemical Reagent Co. Ltd. Water glass with a modulus of 3.3, Baume degree of 40, and composition of 7.9% Na_2_O, 26.2% SiO_2_, and 66% H_2_O by mass was supplied by Shikoku Chemical Corporation. Three activating solutions were prepared by altering the NaOH content in the water glass: low-concentration (L-C), medium-concentration (M-C), and high-concentration (H-C); the chemical compositions and other properties of the solutions are summarized in Table 2.

The basic experiment to explore the use of color variation for determining the hydration kinetics of AAS was conducted with an M-C activating solution. The other solutions (L-C and H-C) were used to investigate the influence of different concentrations of the alkaline activator on the hydration kinetics of AAS, which is discussed in Section 3.5.

A VHX-1000E Digital Microscope from KEYENCE was used to monitor the color variation of AAS in a constant area at 100× magnification over a long period (7 days).

### 2.2. Experimental Methods

#### 2.2.1. Alkali Activation of BFS—Manufacture of an AAS-Sandwich

**Figure 1:**
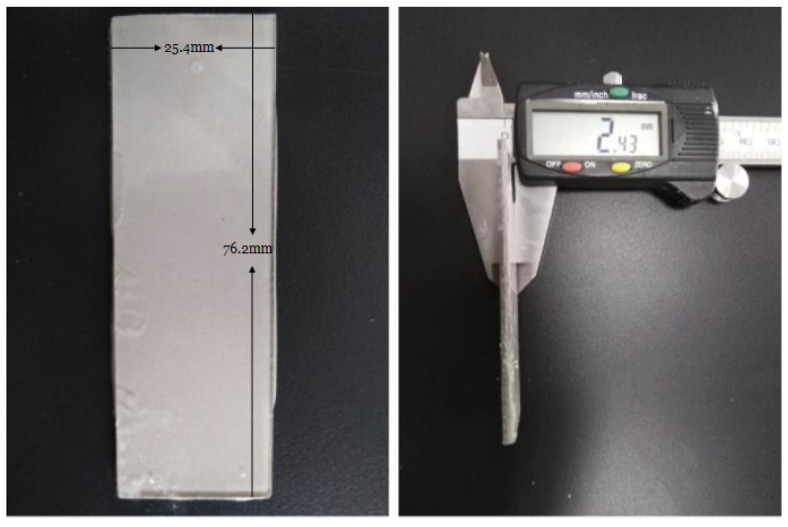
The AAS-sandwich.

The BFS was mixed with the M-C activator described in Table 1 with a BFS:solution mass ratio of 2:1 on a glass slide for 2 min to ensure a thorough mixture of BFS and activator. Another glass slide was placed on top of the slag paste to ensure that the upper surface of the slag paste was at the same horizontal level and prevent water evaporation. The sample was referred to as an “AAS-sandwich.” In this procedure, the thickness of each slag paste between glass slides was set to 2.5 ± 0.1 mm so that the experiments could be easily compared. Furthermore, visible air bubbles were avoided or eliminated on the upper surface of the sample. The AAS-sandwich is shown in Figure 1.

#### 2.2.2. Automatic Continuous Photography of Reaction Products

**Figure 2:**
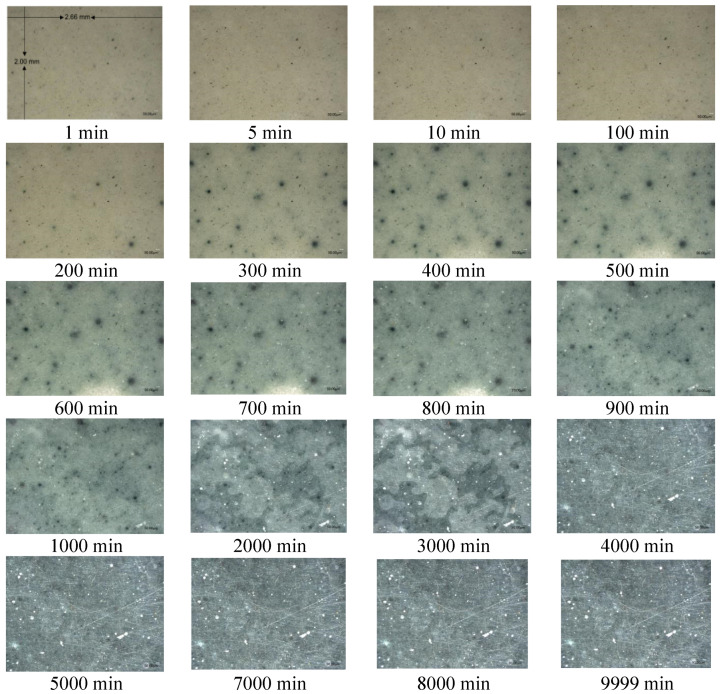
Images of alkaline activated slag (AAS) hydration products acquired at different elapsed times during the reaction.

As soon as it had been prepared, the AAS-sandwich activated with the M-C activator was placed onto the object stage of the VHX-1000E Digital Microscope and photographed. The automatic continuous photography mode was selected to obtain a sequence of images of a fixed area at 100× magnification, and the time interval between each photograph was 1 min. In this study, 9999 images were recorded. Photographs of AAS hydration products (reaction images) acquired at different elapsed times are shown in Figure 2. Figure 2 (1 min) gives the detailed information; the scale bar (50 μm) can be seen at the bottom-right of the photograph, which means the real area of each image frame was 2.66 mm (length) × 2.00 mm (width). This image size was chosen to avoid measurement error caused by variations in hydration degree between small, localized regions.

#### 2.2.3. Digitization of Reaction Product Images

The color of the products of the reaction of BFS with the M-C activator changed from red–gray to blue–green with time as the reaction progressed. The MATLAB software package (MathWorks, Inc., Portola Valley, CA, USA) was used to digitize the color values of all the images to calculate the hydration kinetics: the average pixel value (APV) of each image was computed, and in the subsequent analysis, each image was represented in terms of its three (red, blue, and green) APV values. The resolution of each image was 1600 × 1200 pixels, therefore, the APV was an average of 1,920,000 pixel values. The MATLAB script used is as follows:

filepath = ‘XXX\’;

imgpathlist = dir(strcat(filepath,’*.jpg’));

imgnum = 9999;

if imgnum > 0

r = zeros(1,9999);

g = zeros(1,9999);

b = zeros(1,9999);

end

for i = 1:imgnum

im = imread(strcat(‘VHX_00’,num2str(i),’.jpg’));

s = size(im);

R = im(:,:,1);

G = im(:,:,2);

B = im(:,:,3);

R = reshape(R,s(1),s(2));

G = reshape(G,s(1),s(2));

B = reshape(B,s(1),s(2));

r(1,i) = mean(mean(R));

g(1,i) = mean(mean(G));

b(1,i) = mean(mean(B));

end

where XXX\ is the file path; VHX_00 is the common file name for all the pictures; R, G, and B represent the pixel matrices of the red, green, and blue color channels, respectively; and r, g, and b represent the average pixel values of the red, green, and blue channels of each image.

**Figure 3:**
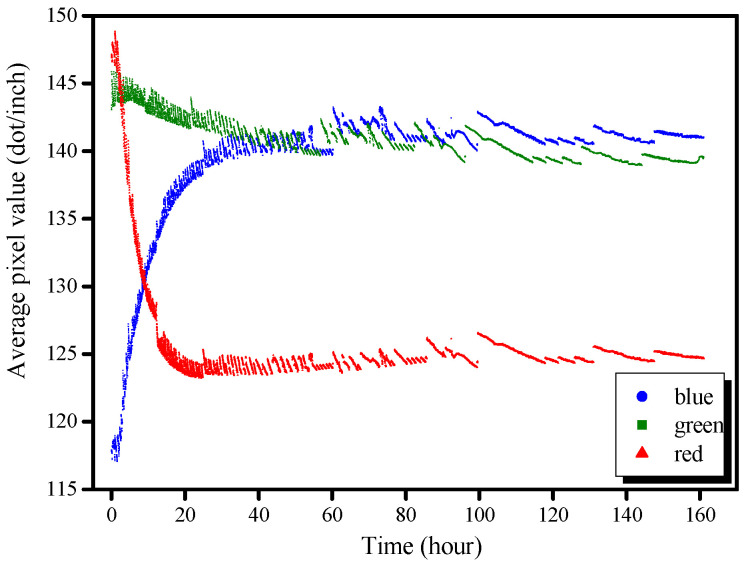
Average pixel values (APVs) of AAS products activated with the medium-concentration (M-C) activator as a function of time.

Figure 3 shows the APVs of red, green, and blue color channels as a function of reaction time, with the data expressed in units of dots per inch. At the beginning of the reaction, the highest APV was red, the lowest APV was blue, and the green APV was slightly lower than the red. The red APV then substantially decreased (approximately 20 h) before remaining constant (20–160 h). The blue APV increased for approximately 30 h and then remained stable. Compared to the other two colors, the variation in the green APV was not significant, and it continuously but gradually decreased with time. This variation was consistent with the visual observation of the AAS product: the color was red-gray at the beginning (Figure 1; 1–100 min), but then rapidly turned green (Figure 1; 300–700 min), and then blue-green (Figure 1; 1000–9999 min).

#### 2.2.4. Smoothing the APV Data

**Figure 4:**
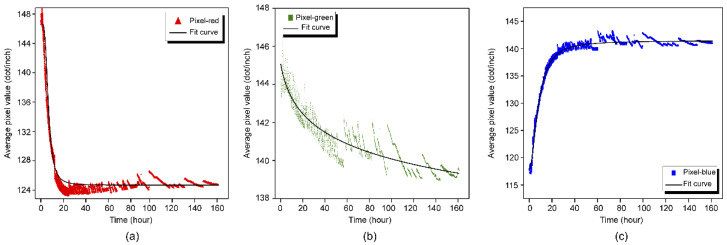
Plots of APV versus time for (**a**) red, (**b**) green, and (**c**) blue channels with functions fitted for smoothing overlaid as black lines.

The APV data were not perfectly smooth because of instrument error related to the use of the VHX-1000E Digital Microscope, which affected the calculation accuracy of the hydration degree (*α*). Therefore, the Origin software package (OriginLab Corporation) was used to fit curves to the plots of the APVs for the three color channels (r, g, and b) versus time. The fitted curves and their equations are shown in Figure 4 and Table 3, respectively.

## 3. Results and Discussion

### 3.1. Reaction Color Variation Velocity v(t) and Color Variation Speed ɛ(t)

Using the equations in Table 3, the color variation velocity and color variation speed, *v*(*t*) and *ɛ*(*t*), respectively, based on the AAS color variation, can be calculated as follows:

(3) $$v_{\left(i\right)}(t)=\left(p_{\left(i\right)}(n){-p}_{\left(i\right)}(n-1)\right)/\bar{t},\;i\in (r,g,b),\;n\in (2,\max)$$

(4) $$\varepsilon_{(i)n}(t)=\left|v_{(i)n}(t)\right|,\;i\in (r,g,b),\;n\in (1,\max-1)$$

where *i* represents the pixel type (red, green, or blue), *p* is the APV fitted in the smoothing process, $\bar{t}$ is 0.01667 h (1 min), and *n* is an index identifying the image.

**Figure 5:**
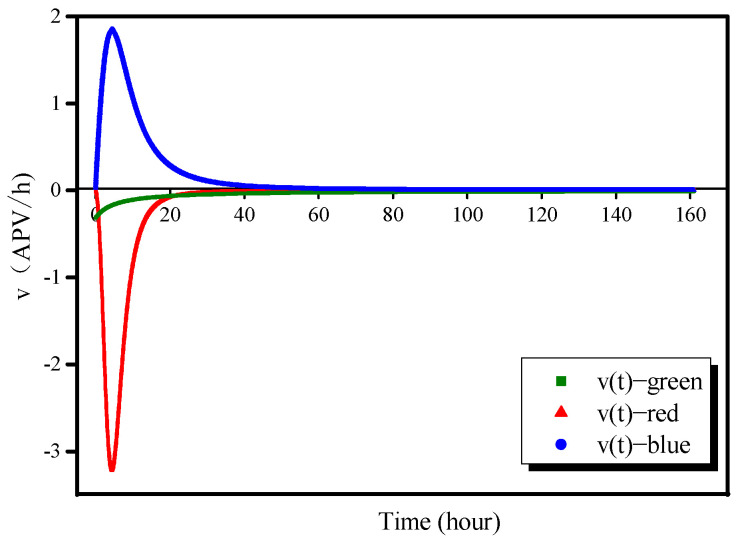
Color variation velocity as a function of time.

**Figure 6:**
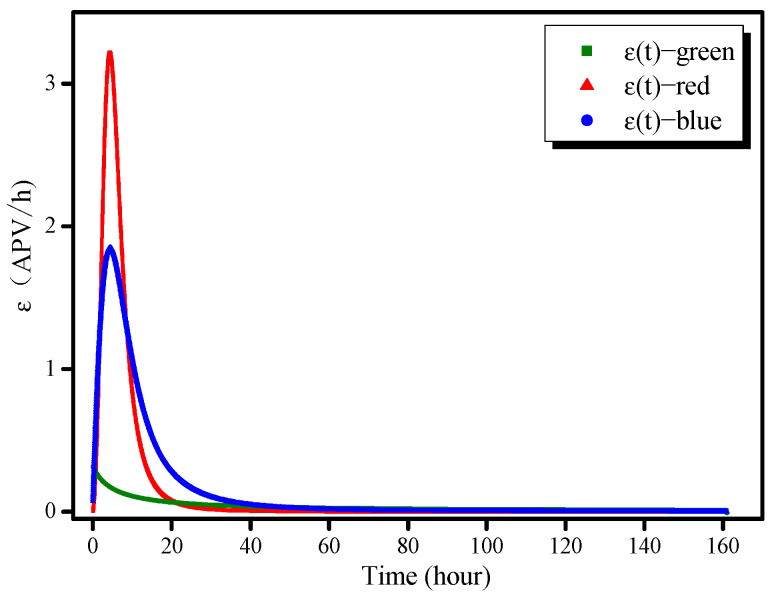
Color variation speed as a function of time.

The color variation velocity *v(t)* and color variation speed *ɛ(t)* represented as functions of time are shown in Figure 5 and Figure 6, respectively. The values of *v*_r_*(t)* and *v*_g_*(t)* are negative, which indicates that the intensities of these two colors are reduced as the reaction proceeds. The red intensity decreases rapidly: *v*_r_ reached −3.2 APV/h at 4.5 h, shifted rapidly to 0.05 APV/h at 22.5 h, and then remained at a very low level thereafter. The green intensity decreased gradually and continuously: *v*_g_(*t*) decreased to a very low value (<0.05 APV/h) at approximately 20 h. In contrast, *v*_b_(*t*) remained positive throughout, which explains why the color of the AAS gradually became blue. An analysis of the color variation speed demonstrates that the AAS color variation is primarily dominated by the red and blue channels; the green channel has little influence on the variation. *ɛ*_r_(*t*) and *ɛ*_b_(*t*) reached their maximum values almost simultaneously (20 h); however, the color-change process as observed via the blue channel took much longer, approximately 40 h.

### 3.2. Calculation of Hydration Rate α(t)

**Figure 7:**
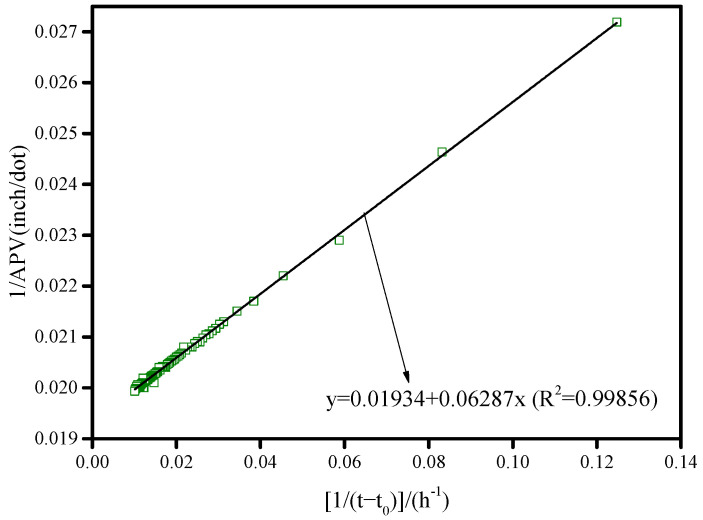
Linear fitting to obtain APV_final_ via extrapolation of the Knudsen equation.

The alkali-activated BFS reaction is a color-change reaction for which the hydration process cannot be described by individual color-channel variation. A comprehensive analysis of the transformation process for all three channels is a feasible method to represent the entire reaction. Therefore, the color variation speed of the entire reaction, *ɛ*(*t*), is defined as the summation of the color variation speed for all three color channels (Equation (5)). The final APV value (APV_final_) is defined as the APV value when time approaches infinity (*t* = ∞); this was calculated as shown in Equation (6) to be 57.1034. The APV_final_ value can be also obtained via extrapolation of the Knudsen equation (Equation (7)) [3], as illustrated in Figure 7; we obtained 57.7063 as the result in this case. Thus, there is little difference between the APV_final_ values calculated using Equations (6) and (7). In this study, the integral method, Equation (6), was ultimately selected as out method of calculating the APV_final_ value. The hydration degree, *α*, is defined in Equation (8), and this was determined by referring to the method reported by De Schutter and Taerwe [18].

(5) $$\varepsilon (t)=\varepsilon_{\left(r\right)}(t)+\varepsilon_{\left(g\right)}(t)+\varepsilon_{\left(b\right)}(t)$$

(6) $${\mathrm{APV}}_{\mathrm{final}}=\int_{t_{0}}^{t_{\infty }}\varepsilon (t)dt$$

(7) $$\frac{1}{{\mathrm{APV}}_{t}}=\frac{1}{{\mathrm{APV}}_{\mathrm{final}}}+\frac{t_{50}}{{\mathrm{APV}}_{\mathrm{final}}\times (t-t_{0})}$$

(8) $$\alpha (t)=\int_{t_{0}}^{t}\varepsilon (t)dt/{\mathrm{APV}}_{\mathrm{final}}=\int_{t_{0}}^{t}\varepsilon (t)dt/\int_{t_{0}}^{t_{\infty }}\varepsilon (t)dt$$

where *ɛ*(*t*) is the AAS reaction rate based on color variation, *t*_0_ is the time at which the photographic acquisition sequence was commenced, *t*_50_ is the time at which the APV values reached 50% of the APV_final_ value, *α*(*t*) is the hydration degree, and *t_∞_* indicates *t* approaching infinity (*t* = ∞).

**Figure 8:**
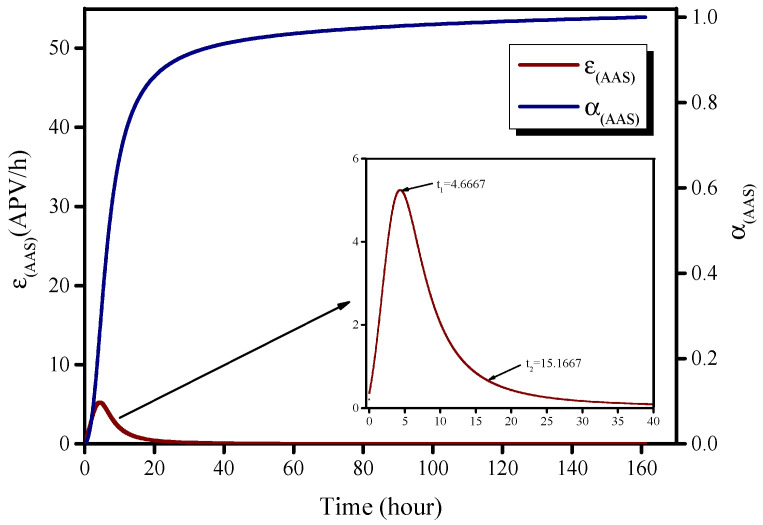
Reaction rate (red) and hydration degree (blue) of AAS based on color variation.

The reaction rate, *ɛ*(*t*), and hydration degree, *α*(*t*), as a function of time are shown in Figure 8, where the blue line denotes the reaction rate, the black line is the first derivative of the reaction rate, and the red line is the hydration degree. The times *t*_a_ and *t*_b_, which are used to deduce the boundaries of different hydration processes in the Krstulovic–Dabic model, are defined when the value of the first derivative of the reaction rate is zero.

The AAS hydration process is characterized by five steps: dissolution, induction, acceleration, deceleration, and a completion period [18]. An isothermal microcalorimeter instrument (e.g., TAM Air) generates two peaks on a plot of reaction rate vs. time. The first peak is generated by the dissolution and induction period and is considered difficult to observe because of the very fast reaction rate; the second peak is associated with the other three steps and is considered valid for the kinetics of the early processes of alkaline activation of BFS. Therefore, only a part of the data produced by an isothermal microcalorimeter instrument can be used to calculate the hydration kinetics, which means that the hydration process studied by this method is incomplete. Similarly, reaction kinetics equations, such as that of the Krstulovic–Dabic model, can only be used to analyze hydration curves with a single peak. By analyzing the color variation, observation of an early hydration process during alkaline activation of BFS can be achieved because only one peak appears in the curve, as shown in Figure 8.

### 3.3. Krstulovic–Dabic Kinetic Model

The alkaline activation of BFS is a complex process in which the BFS structure is first broken down before polycondensation and precipitation of the reaction products occurs. It is generally thought that three basic processes occur in the kinetic model: nucleation and crystal growth (NG), interactions at phase boundaries (I), and the diffusion process (D) [4,7,9,14]. All three processes are believed to occur simultaneously; however, the slowest one dominates the hydration process as a whole, such that it is necessary to determine their rates. On the basis of studies conducted thus far, it has been established that the slowest process at the beginning is NG, but later becomes I or D [1,18]. The basic kinetic equations describing heterogeneous systems are based on the variation in the hydration degree relative to time elapsed (*α–t* data), as shown in Figure 8.

The individual nucleation process occurs at different locations on the particle surface. As these nuclei are randomly dispersed around the particle surface, interactions between them halt their growth; in these cases, the process is controlled by Equation (9) [19]. If the nuclei of the products formed are uniformly distributed on the solid surface, a reacting interphase is rapidly formed, and the process is governed by the phase boundary, as shown in Equation (10) [20]. The equation that best explains this diffusion process is Equation (11) [21]. For most systems, the mechanism is based on the evolution from NG to D.

Nucleation and crystal growth (NG):

(9) $${\left[-\ln(1-\alpha )\right]}^{1/n}=K_{1}t=K_{\mathrm{NG}}t$$

Phase boundary reaction (I):

(10) $${\left[1-{\left(1-\alpha \right)}^{1/3}\right]}^{1}=K_{2}R^{-1}t=K_{I}t$$

Diffusion (D):

(11) $${\left[1-{\left(1-\alpha \right)}^{1/3}\right]}^{2}=K_{3}R^{-2}t=K_{D}t$$

In Equations (9)–(11), *R* is the radius of the reacting particles; *K*_NG_, *K*_I_, and *K*_D_ are the rate constants for the NG, I, and D processes, respectively, and *K*_I_ *= K*_2_*/R*, and *K*_D_ *= K*_3_*/R*^2^. The particle radius has not been considered further.

When *α* is differentiated with respect to *t* in Equations (9)–(11), the rate constants of the three processes are obtained [3,22]. The equations are as follows:

(12) $${\left[\frac{d\alpha }{dt}\right]}_{\mathrm{NG}}=F_{\mathrm{NG}}(\alpha )=K_{\mathrm{NG}}n(1-\alpha ){\left[-\ln(1-\alpha )\right]}^{\frac{n-1}{n}}$$

(13) $${\left[\frac{d\alpha }{dt}\right]}_{I}=F_{I}(\alpha )=K_{I}{\left(1-\alpha \right)}^{\frac{2}{3}}$$

(14) $${\left[\frac{d\alpha }{dt}\right]}_{D}=F_{D}(\alpha )=\frac{3}{2}K_{D}{\left(1-\alpha \right)}^{\frac{2}{3}}/[1-{\left(1-\alpha \right)}^{\frac{1}{3}}]$$

The Krstulovic–Dabic kinetic model [3] is a well-known representative hydration dynamic model, through which the hydration process of inorganic cementing materials can be controlled. Furthermore, the factors affecting each stage can be easily analyzed using this model. However, the model has a number of limitations, including the inclusion of boundaries between the various processes. Furthermore, whether the model divides the hydration reaction process into three basic processes is unclear, and the correlation between the experimental data and models at boundaries is poor. In this study, the Krstulovic–Dabic model was used effectively to simulate the hydration process of AAS.

### 3.4. AAS Hydration Process Simulation

**Figure 9:**
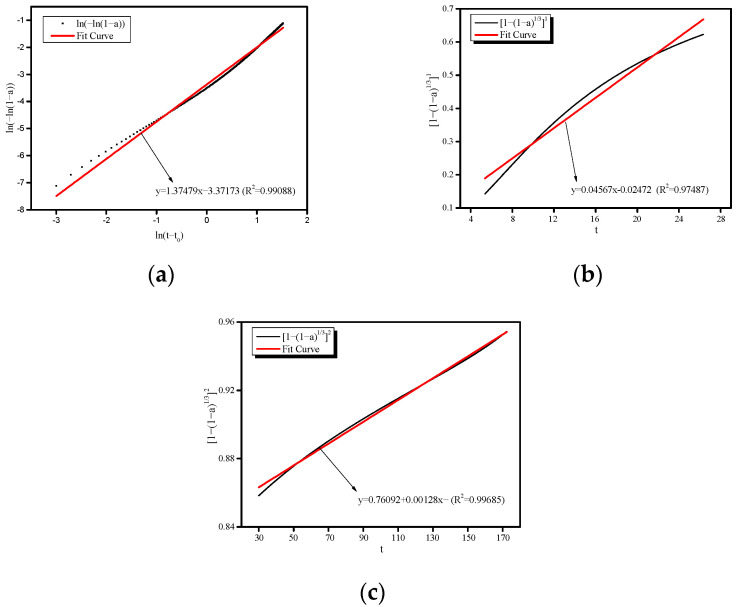
Calculation of *n*, *K_NG_*, *K_I_*, and *K_D_*. (**a**) NG process. (**b**) I process. (**c**) D process.

Through linear fitting, the value for the exponent *n* in the expression for the NG process (Equation (12)) can be determined graphically on the basis of the slope of the straight line from the log–log plot of −ln(1 − *α*) and elapsed time *t*. The value of *n*ln(*K*_NG_) can be determined as an intercept, from which the value of *K*_NG_ can be extracted. The method is illustrated in Figure 9a.

By substituting the hydration degree *α*(*t*) into Equations (13) and (14), the curves of [1 − (1 − *α*)^(1/3)^]/*t* and [1 − (1 − *α*)^(1/3)^]^2^/*t* are obtained, from which the kinetic parameter *K*_I_, the phase boundary reaction (I) parameter and *K*_D_, the diffusion process (D) parameter can be extracted by linear fitting, as shown in Figure 9b,c. The linear fitting process for obtaining *n* and *K* is summarized in Table 4.

**Figure 10:**
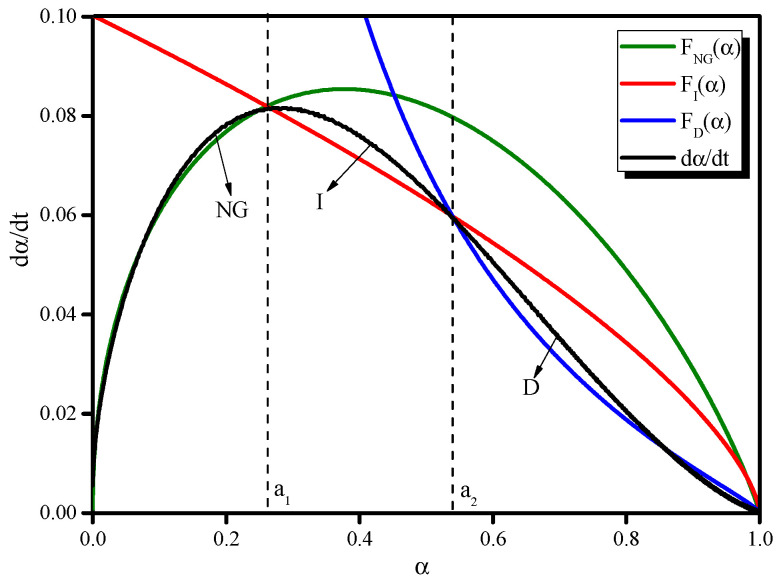
Hydration process for the reaction of BFS with the M–C activator.

The kinetic parameters obtained as described above were substituted into Equations (12)–(14) to retrieve the kinetic curves of the reaction rates, *F*_NG_(*α*), *F*_I_(*α*), and *F*_D_(*α*). To determine the law governing the process of the early hydration of AAS, the relationships between the experimental values and the *F*_NG_(*α*), *F*_I_(*α*), and *F*_D_(*α*) theoretical data corresponding to the hydration degree *α* are shown in Figure 10. The *a_1_* and *a_2_* values can be determined using the intersections of the *F*_NG_(*α*), *F*_I_(*α*), and *F*_D_(*α*) curves shown in Figure 10, where *a*_1_ indicates the transformation point for which the law governing the hydration process changes from NG to I, and *a*_2_ for the change from I to D.

*F*_NG_(*α*), *F*_I_(*α*), and *F*_D_(*α*) can perfectly simulate the actual hydration rate curve, d*α*/d*t*, obtained by measuring AAS color variation. The three theoretical processes (NG, I, and D) are consistent with the experimental curve. Furthermore, the hydration of AAS is not a single process, but rather a complex process with multiple reaction mechanisms that govern different reaction stages. At the beginning of hydration, NG dominates the entire reaction because at this stage, ions migrate easily thanks to there being sufficient liquid and insufficient hydration products in the system. As the hydration proceeds, the reaction is dominated by I and then D because the ion migration ability decreases as the amount of liquid becomes increasingly insufficient [18]. The process sequence is NG–I–D for the M-C activator in this study.

### 3.5. Influence of Alkaline Activator Concentration on the Kinetic Model

**Figure 11:**
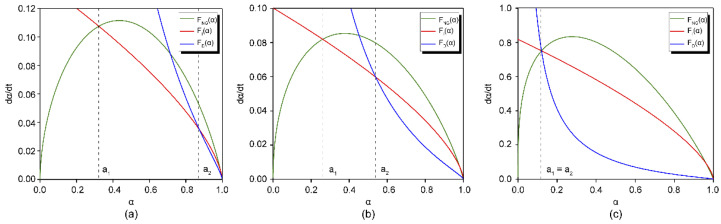
Modeled kinetics for different alkaline activator concentrations: (**a**) L-C activator, (**b**) M-C activator, and (**c**) H-C activator.

In this study, we aimed to confirm whether the reaction kinetics of the entire AAS process can be simulated on the basis of the color variation. The experiment focused on one alkaline activator concentration. Therefore, the influence of the concentration of the alkaline activator (L-C, M-C, and H-C; Table 2) on the kinetics of these processes was also investigated. Figure 11 plots the theoretical expressions of Equations (12)–(14) at different concentrations of alkali activators. The values of the intersections of the theoretical curves correspond to the *a*_1_ and *a*_2_ values, and the fitting of the equation to the experimental values gives the rate constants under different conditions. These values are summarized in Table 5.

Two types of hydration sequences were evaluated for the determination of the reaction process: NG–I–D and NG–D. For the NG–I–D sequence (Figure 11a,b), the process occurs through nucleation until *α_1_*, and this is followed by a phase boundary interaction until α_2_, when a diffusion process starts. For the NG–D sequence (Figure 11c), the reaction occurs through nucleation and diffusion exclusively; *α*_1_ = *α*_2_ is the hydration degree for which the change from one mechanism to another is produced. For the L-C and M-C activators, NG was completed at the intersection point α_1_*,* at which the L-C activator was 0.313 and the M-C activator was 0.262. D started at the intersection point α_2_, at which the L-C activator was 0.871, and the M-C activator was 0.544. L-C had longer NG and I processes compared to M-C. This is because the L-C activator had a low pH value and high water-glass modulus, a slow hydration process, and sparse hydration products with loose structures, which led to a low ionic migration barrier [23]. The difference between *α*_1_ and *α*_2_ was probably a result of the nature and width of the hydration products formed around the BFS grains that remain anhydrous; it varied with the concentration of the alkaline solution [9,18].

For the H-C activator, the three theoretical curves intersected at the same point and a low *α* value (*α*_1_ = *α*_2_ = 0.128; Figure 11c). In other words, with a higher NaOH concentration and lower water-glass modulus, the I process became shorter and vanished under extreme situations. The hydration products accumulated rapidly, and the ionic migration barrier reached a very high value in a short time, making the NG process extremely intense. D began early, and the hydration process directly transitioned from NG to D.

The value of *n*, a parameter indicating the order of the reaction when the hydration mechanism is nucleation (Equation (9)), is also listed in Table 5. Thus, for *n* = 1, the process is described by a first-order equation ($K_{\mathrm{NG}}t=\left[-\ln(1-\alpha )\right]$) [19]. When *n* = 2, the Avrami equation ($K_{\mathrm{NG}}t={\left[-\ln(1-\alpha )\right]}^{1/2}$) describes the process [20]. When *n* = 3, the nucleation process is described by the Erofe’ev equation ($K_{\mathrm{NG}}t={\left[-\ln(1-\alpha )\right]}^{1/3}$) [21]. When the BFS was activated with the H-C solution, a value of *n* = 1.22 was obtained, indicating that the process fit a first-order equation. The *n* values obtained for the BFSs activated with the M-C activator and L-C activator, 1.96 and 2.69, indicate that the nucleation processes could be described by the Avrami and Erofe’ev equations, respectively.

## 4. Conclusions

The method developed in this study facilitates the calculation of kinetic parameters based on the color variation of the AAS hydration process. The entire early hydration process, including the induction period, could be analyzed using this method; this could not be achieved solely using calorimetric data. Three basic hydration processes (NG, I, and D) were clearly differentiated using this model, and kinetic parameters such as *K*, *n*, *α*_1_, and *α*_2_ were also determined. The kinetic models of the activation of BFS using different alkaline solution concentrations were verified using color variation data. Two different process sequences occurred for the hydration reaction of AAS, depending on the concentration of the alkaline activator: NG–I–D or NG–D. NG dominated the entire early process of hydration; however, as the hydration degree increased, I or D became dominant. A high NaOH concentration resulted in an intense NG and an early and extended D.

## Figures and Tables

**Table 1:** Composition of the blast furnace slag (BFS) (wt.%; LOI—loss on ignition at 1000 °C).

CaO	SiO_2_	Al_2_O_3_	Fe_x_O_y_	MgO	MnO	SO_2_	Others	LOI
41.1	32.26	14.69	2.06	6.19	0.29	0.82	2.24	0.35

**Table 2:** Chemical compositions and properties of activating solutions.

Solution	Water Glass (g)	NaOH (g)	H_2_O (g)	Chemical Composition (wt.% of Oxides)			Modulus (SiO_2_/Na_2_O)
				Na_2_O	SiO_2_	H_2_O	
L-C	5	0.1	10	3.3	8.7	88.0	2.8
M-C	5	0.5	10	5.7	8.5	85.8	1.7
H-C	5	1	10	8.7	8.2	83.1	1.1

**Table 3:** Equations fitted for the smoothing process, where *y* is the APV and *x* is time.

Pixel Type	Fitting Equation	*R* ^2^
Red	$y=124.60033+{22.12705/\left[1+(\frac{x}{5.63675}\right)}^{2.89794}]$	0.96486
Green	$y=135.11352+{10.30075/\left[1+(\frac{x}{88.89702}\right)}^{0.61135}]$	0.83748
Blue	$y=141.46698+{\left(-23.57807\right)/\left[1+(\frac{x}{8.13164}\right)}^{1.91985}]$	0.97808

**Table 4:** Detailed information on the linear fitting process.

Process ^1^	Equation	Intercept	Slope	*K*	*n*	*R* ^2^
NG	ln[−ln(1 − *α*)] = *n*ln(*K*_NG_) + *n*ln(*t*)	−3.37173	1.37479	0.086	0.14	0.99088
I	[1 − (1 − *α*)^(1/3)^]^1^ = *K*_I_ × *t*	−0202472	0.04567	0.046	-	0.97487
D	[1 − (1 − *α*)^(1/3)^]^2^ = *K*_D_ × *t*	0.76092	0.00128	0.001	-	0.99685

^1^ NG, nucleation and crystal growth (NG); I, phase boundary reaction; D, diffusion process

**Table 5:** Hydration kinetics parameters for different alkaline activator concentrations.

Solution	*n*	*K*_NG_	*K*_I_	*K*_D_	*α*_1_	*α*_2_	Kinetics ^1^
L-C	2.69	0.168	0.028	0.004	0.313	0.871	NG–I–D
M-C	1.96	0.086	0.046	0.002	0.262	0.544	NG–I–D
H-C	1.22	0.097	0.107	0.001	-	0.128	NG–D

^1^ NG, nucleation and crystal growth (NG); I, phase boundary reaction; D, diffusion process

## Data Availability

The data presented in this study are available on request from the corresponding author.

## Sample Availability

Samples of the compounds are not available from the authors.
